# *Thaptomys* Thomas 1915 (Rodentia, Sigmodontinae, Akodontini) with karyotypes 2n = 50, FN = 48, and 2n = 52, FN = 52: Two monophyletic lineages recovered by molecular phylogeny

**DOI:** 10.1590/S1415-47572010005000053

**Published:** 2010-06-01

**Authors:** Karen Ventura, Maria José de Jesus Silva, Yatiyo Yonenaga-Yassuda

**Affiliations:** 1 Departamento de Genética e Biologia Evolutiva, Instituto de Biociências, Universidade de São Paulo, São PauloSP. Brazil; 2 Laboratório de Ecologia e Evolução, Instituto Butantan, São PauloSP. Brazil

**Keywords:** Atlantic Rainforest, *cytochrome b*, endemism, molecular phylogeny, *Thaptomys*

## Abstract

A novel karyotype with 2n = 50, FN = 48, was described for specimens of *Thaptomys* collected at Una, State of Bahia, Brazil, which are morphologically indistinguishable from *Thaptomys nigrita*, 2n = 52, FN = 52, found in other localities. It was hence proposed that the 2n = 50 karyotype could belong to a distinct species, cryptic of *Thaptomys nigrita*, once chromosomal rearrangements observed, along with the geographic distance, might represent a reproductive barrier between both forms. Phylogenetic analyses using maximum parsimony and maximum likelihood based on partial cytochrome *b* sequences with 1077 bp were performed, attempting to establish the relationships among the individuals with distinct karyotypes along the geographic distribution of the genus; the sample comprised 18 karyotyped specimens of *Thaptomys*, encompassing 15 haplotypes, from eight different localities of the Atlantic Rainforest. The intra-generic relationships corroborated the distinct diploid numbers, once both phylogenetic reconstructions recovered two monophyletic lineages, a northeastern clade grouping the 2n = 50 and a southeastern clade with three subclades, grouping the 2n = 52 karyotype. The sequence divergence observed between their individuals ranged from 1.9% to 3.5%.

Although many of the recently proposed redefinitions of generic groupings within the rodent subfamily Sigmodontinae were based on phylogenetic analyses using molecular markers (Smith and Patton, 1991, 1993, 1999, 2007; D'Elía 2003; D'Elía *et al.*, 2003), much of the debate regarding its taxonomy and systematics has been historically focused on less inclusive taxonomic categories, based on morphological characters (Reig, 1987). This situation is especially true regarding the establishment of some taxa which are morphologically similar to *Akodon* (*e.g.*, *Deltamys, Hypsimys, Microxus, Thaptomys* and *Thalpomys*).

The genus *Thaptomys* Thomas, 1915 was described to allocate *Hesperomys subterraneus* Hensel, 1873 (*e.g.*, *Mus nigrita* Lichtenstein, 1829). Massoia (1963) clarified the identity of *H*. *subterraneus*, *Thaptomys* type-species, as a synonym of the previously named *M*. *nigrita*, reporting a larger area of occurrence for the species. This taxon has been usually referred to as a subgenus of *Akodon* (Ellerman, 1941; Cabrera, 1961) or even as a full synonym of the nominate subgenus (Reig, 1987). In taxonomic reviews, Hershkovitz (1990, 1998) has reasserted the singular morphological traits of *Thaptomys* and assigned the generic status in accordance to Thomas (1915).

Molecular systematic studies carried out by Smith and Patton (1999), using cytochrome *b* sequences, provided evidence from a phylogenetic perspective for the genus *Thaptomys* and led to the conception that the tribe Akodontini is formed by *Akodon* (including *Deltamys,Microxus* and *Hypsimys*)*, Thaptomys, Necromys* (referred to as *Bolomys*)*, Oxymycterus, Lenoxus, Blarinomys, Brucepattersonius, Podoxymys, Juscelinomys, Thalpomys, Scapteromys, Kunsia* and *Bibimys.* So far, *Thaptomys* is a monotypic genus (Musser and Carleton, 2005), containing the species *T. nigrita*, distributed along the Brazilian east coast, eastern Paraguay and northeastern Argentina. Karyotyped samples with diploid number (2n) = 52, fundamental number of autosomal arms (FN) = 52, are recorded for different localities of the Brazilian Atlantic Rainforest in the States of Rio Grande do Sul, Paraná, São Paulo, Rio de Janeiro and Espírito Santo (reviewed in Ventura *et al.*, 2004).

Ventura *et al.* (2004) described a new karyotype with 2n = 50, FN = 48, for specimens of *Thaptomys* sp. (TSP) collected at Una, State of Bahia, Brazil, which are morphologically indistinguishable from *T. nigrita* (TNI) with 2n = 52, found in other Brazilian localities. G-banding patterns and interstitial telomeric signals (ITS) detected by fluorescent *in situ* hybridization (FISH) suggested that the differentiation of the diploid number between both karyotypes is due to a tandem rearrangement involving pairs TNI 2, TNI 24, and pair TSP 2. It has been hence proposed that this new karyotype with 2n = 50 could belong to a distinct species, cryptic of *Thaptomys nigrita*. The chromosomal rearrangements could promote errors in meiotic drive and, along with the geographic distance, might represent reproductive barriers between both forms*.*

Molecular phylogenetic analyses using maximum parsimony and maximum likelihood, based on partial cytochrome *b* sequences of 18 karyotyped *Thaptomys* specimens with karyotypes 2n = 52 and 2n = 50 from eight different localities of the Atlantic Rainforest (Table 1 and Figure 1), were performed, attempting to establish the relationships among the individuals along the geographic distribution of the genus. Samples of *Brucepattersonius*, *Necromys* and *Thalpomys* were used as outgroups. Sequences from GenBank and their accession numbers are listed in parentheses in the cladograms.

Genomic DNA was extracted from liver or muscle samples preserved in alcohol or at -80 °C, following the protocol described by Fetzner (1999). Fragments were amplified using primers MVZ 05 or MVZ 127 for the light-strand, and MVZ 16 or MVZ 14 for the heavy-strand (Smith and Patton, 1993, 1999). Polymerase chain reaction (PCR) was performed in a final volume of 25 μL for each sample, as follows: 4.0 μL of dNTPs (5 mM); 2.5 μL of 10x buffer; 2.5 μL of MgCl_2_ (25 mM); 2.0 μL of each primer; 9.5 μL of milli-Q water; 0.5 μL of Recombinant *Taq* DNA Polymerase (5 U/μL), and 2.0 μL of DNA template. The PCR thermocycling protocol consisted of an initial denaturation step of 94 °C for 5 min, followed by 40 cycles at 94 °C for 30 s, 48 °C for 45 s, 72 °C for 1 min, and a final extension step at 72 °C for 10 min.

The PCR products were directly purified with ExoSAP-IT kit (USB Corporation Biosciences), following the manufacturer's instructions. The sequencing reactions were performed using the ABI PRISM® Big Dye Terminator v. 3.0 kit (Applied Biosystems). Samples were sequenced with the automatic ABI PRISM® 3700 DNA Analyzer (PE Applied Biosystems Foster City, CA, USA).

Light and heavy strands were edited, manually aligned, and compared using Sequence Navigator (Applied Biosystems). Multiple alignments were done with Clustal X. The reading frame of the obtained sequences was inferred using MacClade.

Phylogenetic analyses were carried out by means of the maximum parsimony (MP) and maximum likelihood (ML) methods, using PAUP* 4.0b10 (Swofford, 2001). The MP analysis was performed by a heuristic search using stepwise addition with rearrangement algorithm TBR (tree bisection and reconnection) and 10,000 replicates. The ML analysis was made using the appropriate nucleotide substitution model, selected by Modeltest (version 3.06, Posada and Crandall, 1998); the parameters of this model were used to reconstruct the phylogeny through a heuristic search by stepwise addition with TBR and 1,000 replicates.

The robustness of nodes was assessed by nonparametric bootstrap percentages (Felsenstein, 1985) after 1,000 (MP) and 500 (ML) pseudoreplicates. Bremer branch support (Bremer, 1994) was implemented in the MP analysis.

Fifteen haplotypes were recognized in the sample; specimens from the State of São Paulo UNIBAN2132, UNIBAN2031 and CIT1667 shared haplotype H9, and CIT175 and CIT323 shared haplotype H12 (Table 1).

The matrix generated from all the sequences presented in Table 1 has 1077 bp, with 783 bp constant, 87 bp uninformative and 207 bp informative sites in the parsimony analysis. The MP analysis recovered five most parsimonious trees with a length of 416 steps, a consistency index (CI) of 0.82, and a retention index (RI) of 0.83. Figure 2A presents the strict consensus tree with the respective branch support values*.*

For the ML analysis, the most adequate model as revealed by Modeltest 3.06 (Posada and Crandall, 1998) was the GTR+I+G model (General Time Reversible, Rodríguez *et al.* 1990). The likelihood tree obtained and the respective bootstrap values are depicted in Figure 2B.

Both methods of tree reconstruction resulted in a clade containing the *Thaptomys* lineages (clade Ô) with bootstrap supports of 100% (Figures 2A and B), indicating monophyly of the genus. A basal dichotomy splits *Thaptomys* in two: lineages with 2n = 50 (northeastern), restricted to Una, BA, and with 2n = 52 (southeastern), distributed over the eastern and southern Atlantic Rainforest.

The southeastern clade presented three subclades by both reconstruction methods (Figure 2). Clade S_1_ groups specimens from Santa Teresa and Domingos Martins in the State of Espírito Santo; S_2_ includes specimens from Biritiba-Mirim, São Bernardo do Campo and Pilar do Sul, in the State of São Paulo; S_3_ groups specimens from Iguape and Ortigueira, in the States of São Paulo and Paraná, respectively. By the MP method, the relationship among these three lineages was unresolved, while ML analysis placed S_3_ as the sister group of the clade formed by S_1_ and S_2_.

Sequence divergence between individuals from the northeastern and southeastern clades ranged from 1.9% to 3.5%. Within the clades, sequence divergence varied from 0.2% to 1.1% in the northeastern clade, and from 0.1% to 2.1% in the southeastern clade. Variation found among specimens from the same geographic region was lower than 1% (Table 2).

The cladogram obtained by molecular phylogenetic analyses shows that cladogenesis events in *Thaptomys* are coincident with the presence of rivers (A) Pardo and Jequitinhonha, (B) Doce, (C) Paraíba do Sul and (D) Ribeira de Iguape, and the Serra de Paranapiacaba mountain chain (Figure 1).

The intra-generic relationships recovered by the maximum parsimony (MP) and maximum likelihood (ML) analyses corroborated the distinct diploid numbers, since the 2n = 50 and 2n = 52 karyotypes appeared as distinct monophyletic lineages, sister-group to each other, with the Pardo and Jequitinhonha river systems and Doce River basin as putative geographic barriers involved in their cladogenesis, and also isolated by the tandem chromosomal rearrangement and by the distance determined by their own geographic distribution (Figures 1  and 2; Table 2), which may represent reproductive barriers for the two forms.

Molecular analyses showed two geographically distinct lineages in the Atlantic Rainforest, allowing the identification of two broad regions in this area. Previous studies involving different groups of vertebrates also recognized two broad regions in the Atlantic Rainforest, separating the fauna into northeastern and southeastern components, as demonstrated for lizards (Vanzolini, 1988; Pellegrino *et al.*, 2005), birds (Bates *et al.*, 1998; Cabanne *et al.*, 2007), and non-flying small mammals (Costa *et al.*, 2000; Costa, 2003).

The herein inferred sister-group relationship between the northeast and southeast components was found in three of these examples: the marsupial *Metachirus nudicaudatus* (Costa, 2003), the lizard *Gymnodactylus darwinii* (Pellegrino *et al.*, 2005), and the bird *Xiphorhynchus fuscus* (Cabanne *et al.*, 2007). Pellegrino *et al.* (2005) attributed this disjunct pattern to the presence of the Doce River acting in a process of vicariance.

The hypothesis that the karyotype with 2n = 50 might belong to a new species, cryptic of *Thaptomys nigrita* with 2n = 52, has already been proposed, based on cytogenetic data and sampled localities with two kinds of cytotypes (Ventura *et al.*, 2004). The lineages recovered with molecular data seem to present a disjunct distribution, with no overlapping areas, since the northernmost recorded karyotyped sample of *Thaptomysnigrita* (2n = 52) is from Santa Teresa, ES, and *Thaptomys* sp. (2n = 50) is endemic of Una, BA, further north, which corroborates the hypothesis of Ventura *et al.* (2004).

Cytogenetics along with molecular phylogenetics and geographic distribution data suggest that *Thaptomys* sp. 2n = 50 is a species distinct from *Thaptomysnigrita*, 2n = 52, awaiting proper taxonomic review. The present data show that *Thaptomys* is a more diverse than previously assumed and highlight the idea that the Atlantic Forest has been an important geographic region in the diversification of the sigmodontines, as it harbors unique Sigmodontinae lineages (*e.g.,Thaptomys*, *Blarinomys* and *Brucepattersonius*), as pointed out by Smith and Patton (1999).

The multidisciplinary approach taken herein reinforces the importance of chromosome description in the discovery and characterization of new taxonomic entities. The molecular analyses corroborated chromosomal surveys, indicating that cytochrome *b* is a good marker for characterizing the diversity at the species level within this group of rodents.

**Figure 1:**
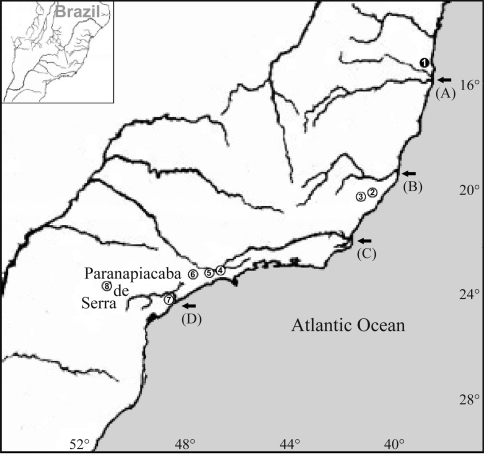
Map of localities of *Thaptomys* specimens with 2n = 50, FN = 48, and 2n = 52, FN = 52, used in the present study. Arrows indicate from north to south the (A) Pardo and Jequitinhonha River system, (B) Doce River, (C) Paraíba do Sul River, and (D) Ribeira de Iguape River. Localities: ❶ Una, BA; ② Santa Teresa, ES; ③ Domingos Martins, ES; ④ Biritiba Mirim, SP; ⑤ São Bernardo do Campo, SP; ⑥ Pilar do Sul, SP; ⑦ Iguape, SP; ⑧ Ortigueira, PR.

**Figure 2:**
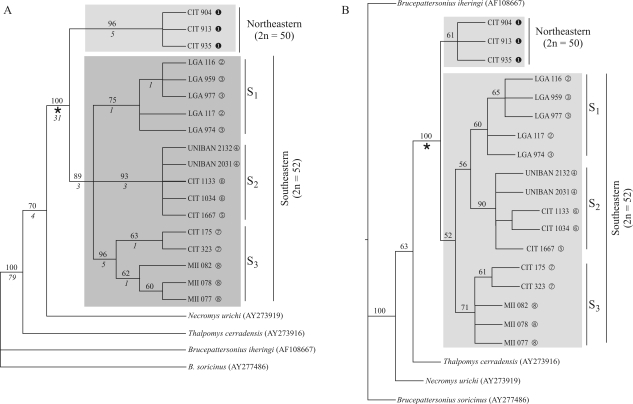
Phylogenetic reconstructions for 18 *Thaptomys* specimens, besides outgroups. A) Strict consensus obtained from five most parsimonious trees (length = 416 steps, CI = 0.82 and RI = 0.83) resulting from 10000 replicates by the MP method. Numbers above nodes represent bootstrap values obtained with 1000 replicates; numbers under nodes are Bremer support values. B) Most likely tree obtained with 1000 replicates by the ML method. Numbers on clades represent bootstrap values obtained with 500 replicates. Access codes of sequences from GenBank are indicated between parentheses. u to q represent localities of each specimen on the map (Figure 1).

## Figures and Tables

**Table 1:** *Thaptomys* samples used in the present study, with their respective identification numbers, localities, karyotypes and haplotype numbers.

Genus	Species	Identification number	Locality	UF^1^	LN^2^	Geographic coordinates	Karyotype	mtDNA haplotype
*Thaptomys*	sp*.*	CIT 904	Una	BA	❶	15°29' S; 39°07' W	2n = 50, FN = 48	H1
		CIT 913					2n = 50, FN = 48	H2
		CIT 935					2n = 50, FN = 48	H3
*Thaptomys*	*nigrita*	LGA 116	Santa Teresa	ES	②	19°56' S; 40°36' W	2n = 52, FN = 52	H4
		LGA 117					2n = 52, FN = 52	H5
		LGA 959	Domingos Martins	ES	③	20°21' S; 40°39' W	2n = 52, FN = 52	H6
		LGA 974					2n = 52, FN = 52	H7
		LGA 977					2n = 52, FN = 52	H8
		UNIBAN 2031	Biritiba Mirim	SP	④	23°57' S; 46°03' W	2n = 52, FN = 52	H9
		UNIBAN 2132					2n = 52, FN = 52	H9
		CIT 1667	São Bernardo do Campo	SP	⑤	23°69' S; 46°56' W	2n = 52, FN = 52	H9
		CIT 1034	Pilar do Sul	SP	⑥		2n = 52, FN = 52	H10
		CIT 1133					2n = 52, FN = 52	H11
		CIT 175	Iguape	SP	⑦	24°42' S, 47°33' W	2n = 52, FN = 52	H12
		CIT 323					2n = 52, FN = 52	H12
		MII 077	Ortigueira	PR	⑧	24°12' S, 50°56' W	2n = 52, FN = 52	H13
		MII 078					2n = 52, FN = 52	H14
		MII 082					2n = 52, FN = 52	H15

^1^Abbreviations of Brazilian states: BA = Bahia; ES = Espírito Santo; PR = Paraná; SP = São Paulo. ^2^Locality numbers as represented on the map (Figure 1) and cladograms (Figure 2).

**Table 2:** Upper diagonal: matrix of corrected genetic distance between *Thaptomys* specimens.

	u	u	u	k	k	l	l	l	m	m	n	o	o	p	p	q	q	q
u	0	0.002	0.008	0.027	0.027	0.029	0.028	0.028	0.029	0.025	0.024	0.028	0.031	0.025	0.022	0.030	0.028	0.030
u		0	0.011	0.025	0.025	0.027	0.026	0.026	0.027	0.023	0.023	0.026	0.029	0.022	0.020	0.028	0.027	0.029
u			0	0.029	0.029	0.031	0.030	0.030	0.027	0.019	0.019	0.019	0.027	0.028	0.025	0.034	0.033	0.035
k				0	0.002	0.002	0.003	0.001	0.007	0.006	0.006	0.009	0.009	0.015	0.012	0.015	0.013	0.014
k					0	0.004	0.003	0.003	0.007	0.005	0.005	0.008	0.009	0.014	0.011	0.014	0.013	0.014
l						0	0.004	0.003	0.009	0.008	0.008	0.011	0.011	0.016	0.013	0.017	0.015	0.016
l							0	0.004	0.008	0.006	0.006	0.009	0.010	0.015	0.012	0.015	0.014	0.015
l								0	0.008	0.006	0.006	0.009	0.010	0.015	0.012	0.015	0.014	0.015
m									0	0.000	0.000	0.003	0.002	0.018	0.016	0.017	0.018	0.018
m										0	0.000	0.003	0.001	0.016	0.016	0.018	0.016	0.018
n											0	0.003	0.001	0.016	0.016	0.017	0.016	0.017
o												0	0.001	0.019	0.019	0.021	0.019	0.021
o													0	0.019	0.017	0.019	0.020	0.020
p														0	0.000	0.004	0.002	0.004
p															0	0.004	0.003	0.004
q																0	0.002	0.003
q																	0	0.001
q																		0

Note: numbers on upper row and left column represent the same localities as on the map (Figure 1).
